# Understanding Opportunity Costs of Women Participating in Maternal and Child Nutrition Interventions in Southern Angola: Evidence From the MuCCUA Trial

**DOI:** 10.1111/mcn.70199

**Published:** 2026-05-21

**Authors:** Rocio Martin‐Cañavate, Antonio Pujol‐de Castro, Chloe Puett, Elena Trigo, Maria de Lourdes Faria, Ana Silva Gerardo, Antonio Vargas, Israel Molina, Estefania Custodio

**Affiliations:** ^1^ Centro Nacional de Medicina Tropical Instituto de Salud Carlos III Madrid Madrid Spain; ^2^ Escuela Internacional de Doctorado Universidad Nacional de Educación a Distancia Madrid Madrid España; ^3^ Department of Family, Population and Preventive Medicine, Program in Public Health, Health Sciences Center Stony Brook University, Stony Brook New York USA; ^4^ Tropical Medicine Unit Vall d'Hebron‐Drassanes, Infectious Diseases Department Vall d'Hebron University Hospital, PROSICS Barcelona Barcelona Spain; ^5^ Fundo Apoio Social‐Local Development Institute Avenida Pedro de Castro Vandunem Luanda Luanda Angola; ^6^ Faculdade de Medicina da Universidade Mandume Ya Ndemufayo, Bairro Comercial Lubango Huíla Angola; ^7^ Action Against Hunger Spain Madrid Madrid Spain; ^8^ Centro de Investigación Biomédica en Red de Enfermedades Infecciosas, (CIBERINFEC), Instituto de Salud Carlos III Madrid Madrid Spain

**Keywords:** Angola, multisectoral nutrition interventions, opportunity cost, societal costs, stunting

## Abstract

Stunting remains highly prevalent in Angola. Multisectoral interventions are essential to prevent this public health issue, and while economic evaluations have assessed the costs and consequences of these interventions, they have largely overlooked costs incurred by participants. The MuCCUA trial evaluates the cost‐effectiveness of three interventions to prevent stunting during the first 1000 days: standard of care (SOC), SOC plus nutritional supplementation (SOC + NS), and SOC plus cash transfers (SOC + CT). This study aimed to estimate participants' opportunity costs and to explore women's experiences. After 12 months of implementation, data were collected from participant women through focus group discussions, interviews and questionnaires on travel and waiting times, and transport costs for attending health posts and community sessions in the SOC arm, and for quarterly distribution visits in the SOC + NS and SOC + CT arms. Total opportunity costs per participant were estimated by intervention arm and community category (close/far from services and/or distribution points). The total opportunity cost of participants over 1 year were US$9373.91 for SOC, US$16,142.09 for SOC + NS and US$18,231.38 for SOC + CT, corresponding to US$1.69, US$2.80 and US$3.17 per participant per month respectively. Value of travel and waiting times accounted for 75%–85% of opportunity costs, with women in remote villages facing substantially higher burdens. These findings highlight the importance of accounting for opportunity costs, especially time and geographic accessibility, when designing maternal and child health and nutrition interventions. Incorporating a societal perspective in economic evaluations is essential to capture unintended participant burdens and promote equitable, effective participation.

**Trial Registration:** NCT05571280. Registered 7 October 2022.

## Summary

- Economic evaluations of multisectoral interventions are critical for stunting prevention, yet most overlook participant‐incurred costs.
- Using MuCCUA trial data, we estimated women's opportunity costs—mainly travel and waiting times, and transport—for three maternal and child interventions in Angola.
- Monthly opportunity cost per participant was $1.69 for standard of care (SOC), $2.80 for SOC+nutrition supplementation and $3.17 for SOC+cash transfer interventions, equivalent to 1–1.5 workdays.
- Opportunity costs were highest for women in remote communities and mostly time‐driven, highlighting the role of geographic accessibility and the need for community‐based interventions closer to populations.
- A societal perspective is essential to capture hidden burdens and ensure equitable and effective programmes.

## Graphical abstract

Economic evaluations of multisectoral nutrition interventions often overlook participants' costs. Using MuCCUA trial data, we estimated women's opportunity costs of three maternal and child nutrition interventions in Angola. Remote participants faced burdens equivalent to 1–1.5 workdays monthly. A societal perspective is essential for equitable, effective and accessible programme design.

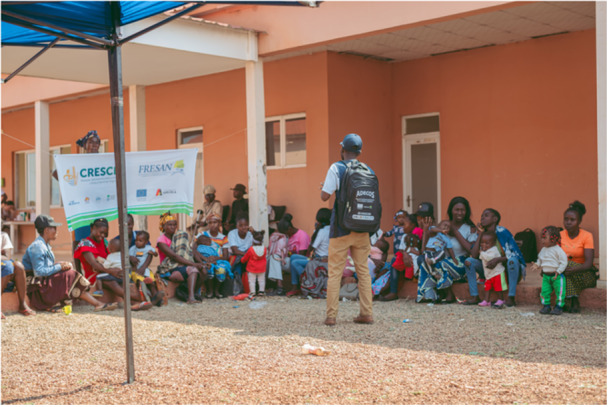

## 1 Introduction

Stunted growth, defined as low height‐for‐age in children under 5 years, is associated with negative lasting physical and cognitive development consequences (Victora et al. 2008). It is a global health burden affecting 150.2 million children (FAO et al. 2025) and grounded on multiple causes including inadequate nutrition, infection, food insecurity, inadequate care, unhealthy conditions, absence of access to health services, social inequalities and poverty, disproportionately affecting low‐ and middle‐income countries (LMICs) (Black et al. 2008). Given the multifactorial nature of stunting, coordinated multisectoral interventions combining nutrition‐specific and nutrition‐sensitive strategies are required (Bhutta et al. 2008). Interventions such as small‐quantity lipid‐nutrient supplements (SQ‐LNS) (Dewey et al. 2021) and cash‐based transfers (Ruel and Alderman 2013) delivered during the first 1000 days of life have been shown to effectively prevent stunting and support optimal child growth (Heidkamp et al. 2021).

Such interventions are often resource‐intensive, making it essential to evaluate the costs at which outcomes are achieved once effectiveness is established. Economic evaluations are particularly important given the scarcity of funding allocated to undernutrition globally (Anfaal et al. 2025). Comparative analyses of alternative strategies, considering costs and consequences, seek to maximize benefits through the efficient allocation of limited resources (Yee et al. 2023). Such decision‐making frameworks are especially critical for multisectoral interventions aiming to prevent child undernutrition in LMICs (Batura et al. 2015; Ramponi et al. 2021; Wun et al. 2022), where trade‐offs between competing options may substantially affect effectiveness, and high costs could threaten feasibility (Njuguna et al. 2020).

Economic evaluations can adopt different perspectives such as institutional, payer or societal, depending on the different sets of costs included (Byford and Raftery 1998; Jönsson 2009; Wun et al. 2022). However, focusing solely on financial costs incurred by institutions provides an incomplete picture (Ramponi et al. 2021; Remme et al. 2017). Few studies adopt a societal perspective or include costs incurred by participants in estimates of total intervention costs (Puett 2019). For example, costs born by families with undernourished children have been largely overlooked, despite these costs often being substantial, including out‐of‐pocket expenses during malnutrition treatment or the opportunity cost of time lost from regular activities while caring for sick children or attending clinic visits (Global Panel 2016; Njuguna et al. 2020).

When designing nutrition and health programmes, it is crucial to anticipate unintended consequences for beneficiaries, including opportunity costs, that refer to the value of the benefits or income that participants forgo by dedicating time and resources to participating in or accessing the intervention, rather than to other productive activities, such as paid work or caregiving (Drummond and Stoddart 1995; Musgrove and Fox‐Rushby 2006). Such opportunity costs are not only major cost‐drivers but also key determinants of service utilization and, consequently, intervention effectiveness (Puett et al. 2013; van den Berg et al. 2006).

This is particularly relevant in rural sub‐Saharan Africa, where women typically shoulder heavy workloads with domestic responsibilities such as cooking, collecting water and firewood and caregiving (Blackden and Q. 2006). In these contexts, multisectoral nutrition interventions targeting women and their children may demand a substantial time investment, potentially placing additional burdens on already busy women and resource‐constrained populations (Johnston et al. 2018; van den Bold et al. 2021). Therefore, considering opportunity costs in economic evaluations is essential to inform the design of more efficient and accessible interventions.

Stunting is a public health concern in Southern Angola, where the Crescer project implements the MuCCUA (Mother and Child Chronic Undernutrition in Angola) community‐based randomised controlled trial, which compares the cost‐effectiveness of three intervention arms combining standard of care, nutritional supplementation and cash transfers (Custodio et al. 2024). Participation in these interventions entails an opportunity cost, an aspect that remains relatively underexplored despite its equity implications (Batura et al. 2015; Naidoo et al. 2020).

Therefore, this paper aims to estimate the costs incurred by participant women and their households as a result of participating in the MuCCUA trial interventions. The specific objectives were: (1) to estimate time spent and transport costs associated with participation in intervention activities, (2) to assess opportunity costs to participants, disaggregated by intervention arm and community category, and (3) to understand participant women's experiences and possible barriers and facilitators for future implementations.

## 2 Methods

### 2.1 Study Setting

The MuCCUA trial was a community‐based, cluster‐randomised controlled trial conducted in Southern Angola between October 2022 and September 2025 which evaluated the effectiveness and cost‐effectiveness of three multisectoral nutrition interventions to prevent child stunting (Custodio et al. 2024). This study was part of the Crescer project, an operational research programme on the prevention of chronic malnutrition in the provinces of Huila and Cunene, funded by the European Union. The project is implemented by a consortium comprising five partner institutions: two in Angola—Mandume Ya Ndemufayo University and the Local Development Instituto‐ FAS—and three in Spain—Hospital Universitari Vall d'Hebron Research Institute, Action Against Hunger Spain and Instituto de Salud Carlos III. The provinces of Huila and Cunene are disproportionately affected by stunting, with prevalence rates of 49.9% and 37.2%, respectively (Governo de Angola, Unicef, World Vision, & Technical Rapid Response Team 2020). The MuCCUA trial was set in these two provinces, including two communes per province—Jamba and Libongue in Huila, and Mupa‐Mukolongondjo and Otchinjau in Cunene—all meeting the inclusion criteria, namely a multidimensional poverty level of 4 or 5 according to the Angola National Statistical Institute classification, approval from local and traditional authorities, and absence of community nutritional interventions in place or forecasted at the time of inclusion. The MuCCUA trial included 36 clusters that were randomized to the three study arms (12 by arm) to enrol 1440 pregnant women. Clusters were defined as villages or neighbourhoods with an approximate population size of around 1075 people. Study participants were pregnant women (≥ 16 years, confirmed by pregnancy test) and their newborns, with the target population for the interventions being the household in which they resided. A total of 1423 pregnant women were recruited and followed‐up with their newborn, until the child reached 2 years of age. The MuCCUA trial protocol and economic evaluation protocol have been published previously (Custodio et al. 2024; Rocio Martin‐Cañavate et al. 2023).

### 2.2 MuCCUA Trial Interventions

#### 2.2.1 Standard of Care Arm (SOC)

The standard of care consisted of two components:

1. Health and nutrition promotion activities implemented by Community and Health Development Agents (ADECOS) that included bimonthly home visits and 1–2 monthly community sensitization sessions aimed at promoting early diagnosis of acute malnutrition through active community screening, vaccination, optimal caregiving and feeding practices and hygiene and sanitation awareness through sensitization sessions.
2. Preventive pharmacological activities included attending health posts for monthly malaria prophylaxis with sulfadoxine–pyrimethamine for pregnant women from the 13th week of gestation; deworming with a single dose of albendazole for pregnant women in the second trimester and to children aged 12–23 months biannually; and vitamin A supplementation semi‐annually to children aged 6–23 months. Pharmacological inputs were supplied to health units and administered by health technicians during prenatal and postnatal consultations based on clinical indication and national protocols.

#### 2.2.2 Standard of Care + Nutritional Supplementation Arm (SOC + NS)

The second arm received the SOC plus nutrition supplementation (SOC + NS) with small‐quantity lipid‐based nutrient supplements (SQ‐LNS) and a family food ration. Individual SQ‐LNS nutritional supplementation consisted of Nutriset Enov' Mum (1 sachet of 20 grs/day) for the pregnant and lactating women until their newborn turns 6 months and Nutriset Enovnutributter + (1 sachet of 20 grs/day) for their newborn children after their 6th month and until the child turns 24 months of age. SQ‐LNS supplements were delivered to participants by ADECOS every 15 days. A complementary family food ration, consisting of a basket of locally produced staple foods, was provided to supplement the usual diet (~ 300 kcal/person/day). The monetary value of the food ration was estimated in 23,781 AOA (US$55.70). The caloric distribution of the basket was 45% of cereals (18 kilograms (kg) of maize flour‐carbohydrate), 30% of legumes (12.5 kg of beans—vegetable protein) and 25% of oil (4 litres of soybean oil—fat), and 1 kg of iodized salt, distributed every 3 months at designated distribution points.

#### 2.2.3 Standard of Care + Cash Transfer (SOC + CT)

Participants in this intervention arm received the SOC plus unconditional cash transfers (SOC + CT). Monthly amounts distributed were 10,855 AOA (US$25.90, October 2022) for households with three or fewer members and 13,855 AOA (US$33.10, October 2022) for households with four or more members. Transfers were delivered directly to study participants on a quarterly basis—equivalent to 33,000 or 42,000 AOA per quarter—at designated distribution points.

Distribution sites for the family food ration and cash transfers were open‐air spaces donated by local communities or government authorities (schools' yards, health posts and communal spaces such as trees or *jangos*).

### 2.3 Data Collection

An economic evaluation was designed including a cost‐efficiency and cost‐effectiveness analysis of the MuCCUA trial interventions from a societal perspective accounting for costs incurred by programme providers, institutions and participants (23).

The current analysis focuses on opportunity costs to participant women in the MuCCUA trial interventions. A total of 1423 pregnant women were recruited in the MuCCUA trial. After 1 year of programme implementation, we collected data to estimate participants' opportunity costs through a field visit undertaken in October 2023. Economic costs were captured through focus group discussions (FGDs) and interviews with a subsample of participants. Different data sources were used to evaluate participants' opportunity costs as well as their perceptions of the programme (Table 1).

**Table 1:** Data sources used in the analyses.

Data source	# Interviews/# FGDs	Topics covered
Beneficiary FGDs	21 × 8–10 participants per group = 186 in total	Costs incurred by beneficiaries, advantages and disadvantages of participation
Key informant interviews with beneficiaries	11	Advantages, disadvantages, additional costs of participation, accessibility and perceived programme impacts
Key informant interviews with community members	40	Locally available wage rates
Household questionnaires	1423	Proportion of women incurring transport costs

Abbreviation: FGD: focus group discussion.

### 2.4 Focus Groups Discussions (FGD)

**Figure 1:**
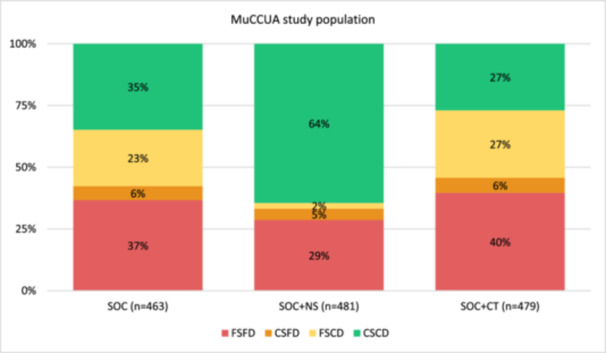
Distribution of MuCCUA trial participants by intervention arm and community category (*n* = 1423). CSCD, Close to services and distribution point; CSFD, Close to services but far from distribution point; FSCD, Far from services but close to distribution point; FSFD, Far from services and distribution point; SOC, Standard of care; SOC + CT, Standard of care plus cash transfer; SOC + NS, Standard of care plus nutrition supplementation.

FGD participants were purposively selected based on their communities' characteristics, evenly distributed across intervention arms to capture variation, and excluded individuals previously selected for interviews. The target study area included 91 villages/neighbourhoods, which were categorized by relevant sociodemographic characteristics including access to health posts and distance to distribution points on foot (Table 2), as reported by ADECOS and supervisors grounded in the community. Villages/neighbourhoods were grouped into four categories: (1) Close to services and distribution point (CSCD), (2) Far from services but close to distribution point (FSCD), (3) Close to services but far from distribution point (CSFD) 4) Far from services and distribution point (FSFD). This selection process aimed to capture diverse beneficiary experiences across geographic areas and service‐access categories. Figure 1 shows the distribution of MuCCUA trial participants by intervention arm and community category.

**Table 2:** Classification of villages/neighbourhoods by community category.

Community category	Description	Distance to health post criteria on foot	Distance to distribution point criteria on foot
CSCD	Close to services and distribution point	≤ 2 h	≤ 2 h
FSCD	Far from services but close to distribution point	> 2 h	≤ 2 h
CSFD	Close to services but far from distribution point	≤ 2 h	> 2 h
FSFD	Far from services and distribution point	> 2 h	> 2 h

*Note:* This categorization was developed based on information from interviews with ADECOS and local supervisors.

Supporting Information S1: Table 1 describes the target population of the MuCCUA trial by community categories. A total of 1423 pregnant women were recruited. The mean age was 26.4% and 47% were younger than 25 years. More than a third of the participants had at least primary education (39%) and being the prevalence of having secondary education was higher in those living in communities close to services (CSCD and CSFD). Overall prevalence of food insecurity measured by low household dietary diversity score was 74.2%, being higher in communities far from services and distribution points (FSFD), (85.5%).

Data on transport costs (direct costs) to and from health posts for the SOC component and to distribution points for the NS and CT components, out of pocket cost, as well as travel and waiting times (indirect costs) for all interventions, were collected based on women's self‐reported estimates. Group discussions were conducted on days when participants were already attending the MuCCUA trial's data collection visits, during which all trial participants were required to attend designated points on three different days for each intervention across the four communes. Project staff were instructed to randomly select participants from the list of participants attending the MuCCUA trial's data collection point to participate in FGDs, based on previously‐mentioned criteria to ensure representation from all community categories and villages/neighbourhoods. This approach was also necessary from a logistical perspective, as conducting separate visits to all participating communities would not have been feasible given the rural and remote nature of the study settings and the need to cover all four communes. In each of the four communes, two trained facilitators conducted FGDs, with one facilitating the discussion and the other providing translation into the local languages. Specifically, FGDs were held in Ganguela, Kioko and Umbundu in the commune of Jamba; Umbundu, Nyaneka‐Humbi and Chokwe in Libongue; Kwanyama in Mupa; and Mungambwe and Mundimba in Otchinjau. A translator rendered the discussions into Portuguese, while a note‐taker documented handwritten notes. In some FGDs, participants also spoke Portuguese, which was used alongside the local languages during discussions. All sessions were audio‐recorded with participants' permission. Personally identifying information of participants, like age or ethnicity, was not included in the selection criteria. Group discussions with participants were chosen as a method for collecting cost data to help ensure they fully understood the questions regarding time spent on various intervention activities. A total of 21 FGDs were performed, 7 by the intervention arm, including 8–10 participants each with a total of 186 participants. Participant women did not receive any financial or in‐kind compensation for participation in the FGDs. Supporting Information S1: Table 2 describes the distribution of FGD participants across interventions and community categories.

### 2.5 Interviews

To complement FGD data, we conducted interviews with a purposive subsample of 1–2 participants from each subgroup and intervention arm to explore perceived advantages, disadvantages, additional costs of participation, accessibility of distribution points, and programme impacts on households and communities. A total of 11 interviews with participants were performed. Project staff were instructed to randomly select participants from the list of participants involved in the MuCCUA trial's data collection phase; excluding those selected for the FGDs. Interviews were assisted by a translator who spoke the local languages and translated participant's responses into Portuguese. A note‐taker documented handwritten notes, and audio was recorded with participants' consent.

In addition, the percentage of women who incurred transportation costs was assessed through survey questions administered to all trial participants during household surveys.

### 2.6 Data Analysis

We estimated opportunity costs using information collected from FGDs and specific survey questions. We divided opportunity costs into two lines: direct costs for transportation (round‐trip travel to and from health post and distribution points) and indirect cost for time spent travelling to and waiting at health posts and distribution points (Supporting Information S1: Table 3). Participants in the SOC + NS and SOC + CT arms also engaged in SOC activities, as all trial arms included the SOC component. Therefore, SOC component opportunity costs were added to opportunity costs of the NS and CT components in those arms.

Micro‐costing data on participants' travel and waiting times and transport costs were summarized and analysed using summary statistics, including mean, median, maximum, and minimum values. Descriptive analyses presented median transportation costs and travel and waiting times to distribution points, community sensitization sessions, and health posts. Time costs were estimated by multiplying the total number of hours spent on intervention‐related activities by the estimated hourly wage. Using a mixed‐methods approach, questionnaire data were used to calculate the proportion of women incurring transport costs per intervention arm and to estimate total transport expenses. Median time and transport costs per participant, by intervention arm and community category, were then extrapolated to all participants by multiplying the time and travel cost by the number of trial participants in each corresponding community category and arm subgroup. Total opportunity costs for each intervention arm were calculated by adding direct and indirect costs.

We estimated the median daily wage for women in the community, with information collected through interviews with community members about the typical daily wages for women in common livelihoods, to value beneficiary's time (Supporting Information S1: Table 4). We assumed a 5‐h working day, as most livelihoods were agricultural labour and used a shadow wage, with an estimated median daily wage of 1500 AOA per day (US$1.91), which was converted into an hourly wage. The median was used to diminish the influence of outliers.

Costs incurred in Angolan kwanzas were converted to 2024 United States dollars (USD) using monthly average exchange rates (24). Microsoft Excel software was used for cost data analysis. Field notes from FGDs and from interviews with participants were translated into English from Portuguese and transcribed in Microsoft Excel. Thematic analysis was conducted by the primary researcher, focusing on participant's perceptions of the programme.

### 2.7 Assumptions

Several assumptions were made to estimate opportunity costs for the first year of interventions implementation, starting with the recruitment of pregnant women at any gestational age. Final estimates were based on clinical guidelines and conversations with project staff and participant women. The following assumptions reflect the intervention activities expected during the first year of implementation.

For the SOC intervention, we estimated the number of health consultations required to receive preventive pharmacological supplies (malaria prophylaxis, deworming and vitamin A intakes). Most participants were in their second (49.7%) or third pregnancy trimester (29.5%), so we assumed two antenatal visits to the health post during pregnancy, followed by one child visit for vitamin A supplementation at 6 months of age. Based on household questionnaires administered to all trial participants, 29% of SOC participants reported incurring transport costs to attend health posts. For the health promotion activities delivered by ADECOS, we assumed participants attended one community sensitization session per month (12 per year). For the SOC + NS and SOC + CT interventions, we assumed four quarterly distributions of inputs (family food ration and cash transfers) per year, and transport costs to attend distribution points were applied to 37% and 43% of participants, respectively, based on the results of the survey data analysis. We assumed minimal variation in participation throughout the year.

### 2.8 Ethics Statement

The research protocol of the MuCCUA trial was approved on August 18th, 2022 by the National Ethics Committee of República de Angola, Ministério da Saúde, reference number 27 C.E/MINSA.INIS/2022, and all methods were performed according to guidelines of the Helsinki Declaration. Additional institutional ethical approval was not sought for the community discussions specifically related to programme participation costs, as these discussions were not deemed to pose additional risk beyond that associated with participation in the main trial. Informed oral consent was obtained from all participants following a clear explanation of the study's objectives.

## 3 Results

During the first year of implementation, the MuCCUA trial interventions reached a total of 1423 pregnant women and their newborns, distributed across study arms as follows: 463 in SOC, 481 in SOC + NS and 479 in SOC + CT.

Notable differences were revealed in median travel and waiting times and transport costs per visit/session across intervention arms and community categories (Table 3). Regarding the SOC intervention, women living in FSFD communities faced the highest transport costs to health posts ($7.7) and the longest round‐trip travel times to health posts with a median of 6.5 h, while women in the CSCD and CSFD groups experienced the lowest costs and shortest round‐trip travel times. In the NS intervention activities, participants in the CSFD and FSFD groups again faced the greatest burdens, with round‐trip travel times to distribution points of 7.0 and 6.0 h, respectively, and median transport costs of $6.4, compared to no transport cost and minimal travel time (0.3 h) in the FSCD group that was far from services but close to the distribution point. Lastly, in the CT intervention activities, participants living in communities close to services but far from the distribution point (CSFD), reported the highest median travel time (11.0 h) and transport cost ($8.9), while women in CSCD and FSCD communities had significantly lower burdens. Additionally, waiting times to receive cash transfers were especially high for the CSFD group (8.0 h), whereas other groups reported shorter waits. Overall, participants in communities far from distribution points (CSFD and FSFD) consistently faced higher direct and indirect costs.

**Table 3:** Summary of transport costs (USD) and time spent (hours) in intervention activities by community category per visit/session.

	CSCD		FSCD		CSFD		FSFD	
	Median (USD [$]/hours)	Range	Median (USD [$]/hours)	Range	Median (USD [$]/hours)	Range	Median (USD [$]/hours)	Range
**Standard of care (SOC)**								
Transport costs to reach health post (USD round trip)	$0.8	($0.3–$2.6)	$1.5	($0.5–$5.1)	$0.00		$7.7	($1.3–$7.7)
Time to the health post (hours on foot round trip)	1	(0.5–3.5)	3	(2.0–6.0)	1	(1.0–2.0)	6.5	(3.0–10.0)
Waiting time to receive medication (hours)	2.5	(0.5–6.5)	4	(1.0–5.5)	1	(0.3–3.0)	1	(0.1–3.7)
Time spent in community sessions (hours)	2	(1.0–3.0)	1	(0.6–2.0)	2	(2.0–2.5)	3.5	(1.0–4.0)
**Nutrition supplementation**								
Transport costs to reach the distribution point (USD round trip)	$2.2	($0.5–$2.6)	$0.0	—	$6.4	(5.1–7.7)	$6.4	($3.8–$10.2)
Time to the distribution point (hours on foot round trip)	2	(1.0–4.0)	0.3	(0.1–0.5)	7	(6.0–8.0)	6	(1.2–18.0)
Waiting time to receive the supplies (hours)	2	(1.0–6.0)	2	—	1	—	2	(1.0–5.0)
**Transport costs**								
Transport costs to reach the distribution point (USD round trip)	$1.3	($1.3–$2.6)	$0.0		$8.9	($2.6–$10.2)	$6.4	($2.6‐$11.5)
Time to the distribution point (hours on foot round trip)	2	(1.0–2.0)	1.1	(0.15–2.0)	11	(4.0–18.0)	8	(4.0–16.0)
Waiting time to receive the supplies (hours)	3	(1.0–5.0)	3	(2.0–4.0)	8	—	2.5	(1.0–8.0)

*Note:* Community categorization was based on staff decisions made with information reported by local staff and community. Values in this table were reported by intervention participants. All costs are in 2024 USD ($). †† “‐“: is used when only one participant provided an estimate or when multiple participants provided a single identical value, making it impossible to calculate a range.

Abbreviations: CSCD, Close to services and distribution point; CSFD, Close to services but far from distribution point; FSFD, Far from services and distribution point; FSCD, Far from services but close to distribution point.

### 3.1 Total Opportunity Costs

The estimated total opportunity cost for interventions participants over the 12‐month implementation period were $9374.91, $16,142.09 and $18,231.38 for the SOC, SOC + NS and SOC + CT interventions, respectively (Table 4). The total opportunity cost per participating woman was $20.25 in the SOC arm, $33.56 in the SOC + NS arm and $38.06 in the SOC + CT arm. Monthly opportunity cost per participant was $1.69 in the SOC, $2.80 in the SOC + NS and $3.17 in the SOC + CT arms, equivalent to between one and one and a half days of work (see Supporting Information S1: Table 5). Women attending distribution points in the SOC + CT and SOC + NS arms faced higher opportunity costs, primarily due to increased direct costs related to travel expenses compared to women in the SOC arm. However, indirect costs including time spent travelling, waiting and attending activities far exceeded direct costs, accounting for 75% (SOC + NS), 77% (SOC + CT) and 85% (SOC) of total opportunity costs. The higher costs observed in the SOC + NS and SOC + CT arms also reflect the inclusion of the SOC component; when assessed independently, the monthly opportunity costs of the NS and CT components alone were $1.11 and $1.48 per participant, respectively.

**Table 4:** Total opportunity cost of participants by intervention arm and community categories.

	SOC (*n* = 463)	SOC + NS (*n* = 481)	SOC + CT (*n* = 479)
	USD [$] (%)	USD [$] (%)	USD [$] (%)
Opportunity costs by cost type			
Indirect costs (time)	$7,965.24 (85%)	$12,170.74 (75%)	$13,952.15 (77%)
Direct costs (transport)	$1,408.67 (15%)	$3,971.35 (25%)	$4,279.23 (23%)
Total opportunity cost	$9,373.91 (100%)	$16,142.09 (100%)	$18,231.38 (100%)
Per participant per year	$20.25	$33.56	$38.06
Per participant per month	$1.69	$2.80	$3.17
Opportunity costs by community category			
Close to services and distribution point (CSCD)			
Per participant per year	$13.91	$29.58	$30.10
Per participant per month	$1.16	$2.47	$2.51
Far from services but close to distribution point (FSCD)			
Per participant per year	$13.98	$23.74	$26.56
Per participant per month	$1.17	$1.98	$2.21
Close to services but far from distribution point (CSFD)			
Per participant per year	$11.48	$41.94	$64.74
Per participant per month	$0.96	$3.50	$5.39
Far from both services and distribution point (FSFD)			
Per participant per year	$31.49	$41.94	$47.32
Per participant per month	$2.62	$3.50	$3.94

*Note:* All costs are in 2024 USD ($). Median daily wage at the community was $1.91.

Abbreviations: SOC, Standard of care; SOC + CT, Standard of care plus cash transfer; SOC + NS, Standard of care plus nutrition supplementation.

### 3.2 Thematic Analysis

Several themes emerged during community discussions and participant interviews.

### 3.3 Accessibility of Distribution Points and Transport Costs

Overall, women living close to distribution points (CSCD and FSCD) reported no accessibility issues, whereas those living in villages far from distribution points (CSFD and FSFD) faced greater challenges, including transport costs that varied by intervention. Participants in the SOC + NS arm reported needing assistance to carry the family food ration.

The distribution point is very far, it takes me 4 h on foot and I always walk because the moto taxi costs 3000 kwanzas one‐way. Distance is a problem for me and my sister has to come with me to collect and carry the food.

Interview #6 with participant woman of the SOC + NS intervention from a FSFD community

It takes 1 hour by motorbike to get to the distribution point, but it is good because it is a middle point for everyone. Going by motorbike to km 27 (distribution point) is 3000 kwanzas in total to come and go.

Interview #4 with participant woman of the SOC + CT intervention from a FSFD community

It takes one hour walking to go to the distribution point, but it is not a problem for me. It didn't generate any costs, only benefits.

Interview #4 with participant woman of the SOC + CT intervention from a FSCD community

I have to spend money on transport to get to the fixed point. I would like transportation to be provided from the villages to here because sometimes I pay for a moto taxi with part of the food I received.

FGD #3 participant woman of the SOC + NS intervention from a FSFD community

In addition, seven women from the SOC + CT intervention mentioned during the FGDs that they go on foot to the distribution point and then return home by moto taxi, using part of the cash transfer to cover the fare, which ranged between 1000 and 2000 kwanzas ($1.28–$2.55), equivalent to approximately 2%–6% of the total quarterly amount received.

#### 3.3.1 Advantages of Participation

According to participant women, the interventions contributed to improved household wellbeing through increased access to food, income and household items allowing them to meet basic needs.

I like the money because it helps us with diseases. We have also bought a pig, goats, clothes, soap, and food (maize flour and oil). Now my children have more support at school. There are 8 of us at home; I have 6 children.

Interview #1 with participant woman of the SOC + CT intervention from a CSCD community

Participating in the programme helps us because we receive food and the papinhas (SQ‐LNS). Now we can buy other things like soap or medicines. We have learnt about hygiene and cooking in the palestras (community sessions) with ADECOS.

Interview #3 with participant woman of the SOC + NS intervention from a CSCD community

Moreover, participants appreciated the counselling and community sessions, reporting that the knowledge gained on child health, hygiene, and nutrition contributed to perceived healthier children with better growth and reduced illness.

I like ADECOS counselling as we learn about child health and vaccines. I see that children are healthier and have more weight.

FGD #8 participant woman of the SOC intervention from a FSCD community

I like the palestras because we learn things we didn't know like that the first milk (colostrum) is a vaccine and good for children. ADECOS are always available and tell us to go to them whenever we have a problem and they accompany us to the health post. Children are growing better, with less diseases and more weight.

FGD #8 participant woman of the SOC + NS intervention from a CSCD community

#### 3.3.2 Disadvantages of Participation

Participant women in the SOC arm reported challenges to participation, expressing dissatisfaction with not receiving cash transfers or food like participants in the other arms. Others in the SOC + CT arm described intra‐household difficulties in managing the cash received.

I would like to receive food as the others do. We are hungry and I don't have money. My family says that I come here and I receive nothing. I lose my time.

Interview #5 with participant woman of the SOC intervention from a FSFD community

One challenge I face is that my husband asks for the money I receive to make business, so not always I keep it or only a little part stays with me.

Interview #1 with participant woman of the SOC + CT intervention from a CSCD community

I think that the cost of the food ration is not equivalent to the money given to women in the cash intervention.

FGD #9 participant woman of the SOC + NS intervention from a CSCD village

Moreover, during FGDs, seven women in the SOC + NS intervention mentioned that their neighbours and other community members complained because only certain households received food rations and that created tensions within communities.

Neighbours speak ill of us and get angry because they do not receive the food ration. I feel that envy exists.

FGD #4 participant woman of the SOC + NS intervention from a FSFD community

## 4 Discussion

Opportunity costs refer to the benefits or income that participants forgo by dedicating time and resources on interventions rather than on other productive activities such as paid work or caregiving. This study presents a detailed estimation of the opportunity costs of women's participation in multisectoral maternal and child nutrition interventions of the MuCCUA trial in rural Angola. Over the 12‐month implementation period, the opportunity cost per participating woman was estimated at $20.25 in the SOC arm, $33.56 in the SOC + NS arm, and $38.06 in the SOC + CT arm. Notably, total monthly opportunity cost per participant was $2.80 in the SOC + NS and $3.17 in the SOC + CT arm equivalent to approximately one and a half days of work. Time spent travelling and waiting was a major driver of opportunity costs representing between 75% and 85% of total costs. Geographical distribution was an important determinant, as participants from distant villages incurred opportunity costs twice as high as those from villages nearby the distribution point.

When considering opportunity costs, it is important to distinguish between direct expenses such as transportation, and indirect costs associated with time. In our study, indirect costs, including time spent travelling, waiting, and participating in activities, represented the majority of the economic burden. This substantial difference between direct and indirect costs aligns with the MAM'Out study in Burkina Faso, which evaluated the cost‐efficiency of a mobile cash transfer to prevent child undernutrition; and found that indirect costs, mainly waiting times (15 min‐8 h) accounted for 85% of beneficiary costs versus 15% for transportation costs (Puett et al. 2018). In our study, waiting times at distribution points compared similarly in the CT arm ranging from 1 to 8 h, highlighting the need for interventions to minimize time demands on participants, and specifically to rationalize choice of distribution point location, in order to improve accessibility and adherence.

Geographic accessibility was also a key determinant of opportunity costs. Participant women living in remote communities far from services and distribution points consistently faced the greatest burdens in all three interventions, with round trip travel times up to 18 h and transport costs representing a substantial share of potential earnings ranging from $2.6–$11.5 (equivalent to 1.4–6.0 days of daily wage). Conversely, those living near services and distribution points had the lowest costs, underscoring how proximity markedly reduced their economic burden. This pattern is consistent with findings from Nepal, where the Suaahara II study found that opportunity costs were highest in the mountain regions due to household dispersion and access constraints such as distance and poor roads (Choo et al. 2024). This recurring trend across interventions highlights the need for rural programmes to integrate accessibility into their design through decentralization, mobile services, or strategically located distribution points, to reduce inequities and improve participation. For instance, the SNACK programme in Mali addressed these challenges by proposing mitigation measures such as covering transportation cost for participants (Le Port et al. 2019).

In our study, opportunity costs varied considerably by intervention arm and community category with total estimated monthly cost per participating woman ranging from $1.7 in the SOC arm to nearly double that, $3.2, in the SOC + CT arm. Given that median estimated daily wages in these rural Angolan communities ranged from $0.29 to $7.33, participation could, in some cases, equate to 11 days of work. This underscores the importance of accounting for these costs, particularly in food insecure and low‐resource settings, where economic barriers especially time and income foregone, may limit access and adherence to interventions (Njuguna et al. 2020). In the MAM'Out study, daily wages were estimated between $0.9 and $6.2 and beneficiary costs at $2.4 per participant per month, equivalent to approximately two and a half days of work per month (Puett et al. 2018). In contrast, the UPAVAN study in India, which evaluated nutrition‐sensitive agriculture interventions through videos, reported lower opportunity costs of $0.68–$0.90 per participant per month for group meetings and home visits, as no travel to distant distribution sites was required (Haghparast‐Bidgoli et al. 2022). Another study in Chad comparing food assistance (FA) with FA plus ready‐to‐use therapeutic foods (RUSF), found higher beneficiary costs of $6.10 in the FA arm and $6.20 in the FA + RUSF arm per child per month, largely due to long distances between distribution sites and the fields, where seasonal agricultural work was taking place during the study period (Puett et al. 2013). However, unlike our study, these estimates were derived from key informant interviews with implementing staff familiar with local travel distances and transportation rates, rather than being collected directly from beneficiaries.

Although opportunity costs can be challenging to measure, they are a fundamental component of economic evaluations taking a societal perspective. In the MuCCUA trial, opportunity costs accounted for 4.7% of total intervention costs in the SOC arm, 3.0% in SOC + NS and 4.2% in SOC + CT (Martin‐Cañavate et al. 2025). Similarly, the PROMIS study in Burkina Faso and Mali, comparing nutrition supplementation strategies, reported opportunity costs of 5.7%‐5.9% of total costs (Brander et al. 2024); while in the MAM'Out study opportunity costs represented 6% of total costs (Puett et al. 2018). In contrast, the SPRING study in Uganda which evaluated different nutrition supplementation approaches, reported lower opportunity costs at 1.9% of total costs (Adams et al. 2023). In this study, some participants incurred food expenses while travelling, though these, as in the MuCCUA study, were negligible and excluded.

In the SOC intervention of our study, participants had to travel to health posts to obtain pharmacological inputs with median roundtrip times of 1.6 h in all categories except FSFD, where it reached 6.5 h. As Musgrove et al. argued, high travel or waiting times can make an intervention less cost‐effective, not due to the intervention itself, but with health facilities that are too distant from beneficiary populations, understaffed or inefficiently managed (Musgrove and Fox‐Rushby 2006). In rural areas, geographic isolation compounded by long‐term social and economic inequities has historically limited access to resources and services resulting in poor nutrition outcomes (Cunningham et al. 2017). Our findings highlight the importance of considering geographic accessibility when selecting distribution sites, and we encourage reporting participant's opportunity costs, disaggregated by distance to health posts and other key service points.

Opportunity costs also increased with intervention complexity being highest in the SOC + NS and SOC + CT arms. Some participants in the SOC + CT intervention reported using part of the cash transfer to cover transport costs, which may have reduced the value of the transfer by $2.2–$5.4 per month representing 6%–20% of the gross total transfer. This suggests that for participants living farther aways from distribution points, the intervention's effect could likely be diminished. Similarly, the REFANI study in Pakistan, which evaluated different cash transfer modalities, estimated opportunity costs at $2.81 representing between 5.6% and 19.2% of the total gross transfer. They highlighted that one reason why cash‐based interventions may appear less cost‐effective for nutrition outcomes than nutrition‐specific interventions, is that sometimes the whole transfer does not reach beneficiaries pocket, as transport expenses absorb part of it (Trenouth et al. 2018). Similarly, some participants in our SOC + NS arm reported using part of the family food rations to pay moto‐taxi rides to return home after distributions.

Finally, thematic analysis complemented our findings. Interviews and FGDs revealed that, although women perceived substantial benefits such as improved child nutritional status, food access, and increased health knowledge, they also faced barriers to sustained participation, including the physical and logistical burden of transporting family food rations and intra‐household management of cash transfers. Another barrier was perceived inequality between intervention arms, with tensions arising between families receiving cash transfers or nutritional supplementation and those who did not. Such perceptions of unequal distribution created tensions within communities, underscoring the risk of social friction when programme benefits are not universally accessible. This was previously reported in a Cochrane systematic review on experiences of recipients of cash transfers programmes intended to improve health outcomes (Yoshino et al. 2023), highlighting the importance of transparent communication, clear eligibility criteria, and community engagement strategies to mitigate these issues (Della Guardia et al. 2022). This occurred in our study, despite the SOC intervention being designed to target and benefit entire communities rather than only trial participants. ADECOS delivered sensitization sessions to all community members, and health units were supplied with preventive pharmacological inputs for all women and children during prenatal and postnatal consultations. Additionally, extensive efforts were made by the Crescer project before and during the trial, to sensitize community leaders, municipal authorities and local communities about the research‐based nature of the study and resulting limited number of beneficiary families in the SOC + NS and SOC + CT arms, intended to implement transparent procedures and to prevent misunderstandings and tensions.

### 4.1 Strengths and Limitations

This study has several limitations. First, opportunity costs estimation relied on data obtained through FGDs and interviews introducing subjectivity and potential recall bias and may not be representative of the total study population. Attrition was higher in the standard‐of‐care arm than in the intervention arms, which may have resulted in selective retention. Consequently, participants who remained engaged in study activities, including FGDs, may not fully represent all women initially enroled, particularly in the standard‐of‐care arm. However, a key strength of our study is that FGDs were purposively designed to include women from diverse community categories and geographic regions within the trial area, resulting in a relatively large sample of 186 participants across 21 FGDs and capturing a broad heterogeneity of perspectives. Further, the FGD tools and setting were also designed to ensure that all participants understood well the questions related to time and costs and gave accurate responses on these questions. Second, opportunity costs were reported in USD, appearing relatively low due to the low wages for unskilled labour in LMICs (Margolies et al. 2021). However, considering actual hours spent waiting or travelling or the equivalent in workdays, as we did in our analysis, provides a more accurate reflection of the magnitude of participants' opportunity costs. At the same time, this study only focused on the opportunity costs incurred by participants when engaging in intervention activities. Potential monetary benefits arising from the interventions, such as reductions in healthcare expenditures, other economic gains associated with improved child health outcomes, or additional benefits gained during visits, such as accessing other health services for themselves or their children or purchasing goods not available locally, were not included in this analysis and may partially offset these costs. However, these aspects will be examined in a forthcoming full economic evaluation once effectiveness data from the MuCCUA trial become available. Another strength of our study was that travel time data from villages to health services or distribution points were collected directly from participants themselves. This contrasts with other studies that relied on official sources, which may be outdated or not reflective of local realities, or that obtained estimates from programme staff, or made assumptions about travel times (Puett et al. 2013; Trenouth et al. 2018). Finally, a key strength of our study was that daily wages to estimate the monetary value of time were obtained and triangulated through interviews with community members allowing us to fully capture the heterogeneity of earnings and employment opportunities.

## 5 Conclusion

Our study provides important insights for the design and evaluation of future community‐based interventions in similar settings, by quantifying women's participation costs, including travel and waiting times and transport costs, and capturing their experiences through a mixed‐methods approach. Women's participation in multisectoral nutrition interventions in Southern Angola entailed substantial opportunity costs, mainly driven by travel and waiting times. These costs were primarily influenced by geographic accessibility, with women in remote communities facing disproportionately higher burdens. Despite these challenges, participant women reported benefits, including improved child health, greater financial flexibility, and increased health knowledge. However, the need to allocate part of these benefits to cover transport costs reduced their net value in the SOC + NS and SOC + CT arms.

Incorporating participants' opportunity costs into economic evaluations is essential to accurately capture the full investment required and to identify programmatic adjustments that could enhance equity and efficiency. It also enables more accurate prediction and measurement of programme results while accounting for the influence of opportunity costs in fragile economies. To strengthen equity and sustainability, future interventions should explicitly incorporate opportunity cost implications in their design. Strategies such as decentralising distribution points, reducing travel and waiting times, strengthening logistical planning and staffing capacity to improve efficiency, integrating food and cash transfer deliveries into routine health services, or providing transportation support could substantially reduce participants' burden and improve adherence to interventions, ultimately maximising their contribution to maternal and child nutrition outcomes.

## Author Contributions

R.M.‐C., E.C. and C.P. conceptualised the costing study, designed the cost data collection tools and the processes for allocating costs. R.M.‐C. collected, curated and analysed cost data. R.M.‐C. and A.P.C. prepared the draft of the manuscript. E.C. and C.P. oversaw the study analysis and reviewed the manuscript. E.C., E.T., M.L.F., A.S‐G., A.V. and I.M. contributed to the conception and design of the MuCCUA trial in which this study is embedded. E.T., M.L.F., A.S.‐G. and I.M. led the supervision of all intervention implementation activities and contributed to field data acquisition. All authors critically reviewed and commented on the manuscript. All authors approved the submitted manuscript.

## Conflicts of Interest

The authors declare no conflicts of interest.

## Supporting information


**Table S1:** Sample characteristics stratified by community category. **Table S2:** Distribution of participants in the population and in the FGDs through arm of intervention and community type. **Table S3:** Description of costs included in opportunity cost by intervention component. **Table S4:** Description of costs included in opportunity cost by intervention component. **Table S5:** Monthly opportunity cost in days of daily wage equivalent.

## Data Availability

The data that support the findings of this study are available from the international project coordinator, Israel Molina Romero, on reasonable request at israel.molina@vallhebron.cat.
