# Supplementary material for: Understanding Opportunity Costs of Women Participating in Maternal and Child Nutrition Interventions in Southern Angola: Evidence From the MuCCUA Trial
Source: Matern Child Nutr. 2026 May 21;22(3):e70199. doi: 10.1111/mcn.70199 (PMC13205709; doi:10.1111/mcn.70199)
Supplement: Supplementary file 1 — Table S1: Sample characteristics stratified by community category. Table S2: Distribution of participants in the population and in the FGDs through arm of intervention and community type. Table S3: Description of costs included in opportunity cost by intervention component. Table S4: Description of costs included in opportunity cost by intervention component. Table S5: Monthly opportunity cost in days of daily wage equivalent. [file MCN-22-e70199-s001.docx]

**SUPPLEMENTARY MATERIAL**

**Supplementary Table 1. Sample characteristics stratified by community category**

|  | **CSCD** | **FSCD** | **CSFD** | **FSFD** | **Overall** |
| --- | --- | --- | --- | --- | --- |
|  | **N=600** | **N=248** | **N=77** | **N=498** | **N= 1423** |
| **Individual level characteristics** |  |  |  |  |  |
| Women age |  |  |  |  |  |
| 15-24 | 48,8 | 54,8 | 40,8 | 43,2 | 47,5 |
| 25-34 | 36,6 | 32,7 | 39,5 | 37,5 | 36,4 |
| 35-49 | 14,6 | 12,5 | 19,7 | 19,3 | 16,1 |
| Education |  |  |  |  |  |
| No education | 44,3 | 43,9 | 41,5 | 58,2 | 49,0 |
| Primary | 37,7 | 47,2 | 42,9 | 36,8 | 39,3 |
| Secondary or higher | 18,0 | 8,9 | 15,6 | 5,0 | 11,7 |
| **Household level characteristics** |  |  |  |  |  |
| Sex of head of household |  |  |  |  |  |
| Male | 61,0 | 77,3 | 74,3 | 71,3 | 68,2 |
| Female | 39,0 | 22,7 | 25,7 | 28,7 | 31,8 |
| Head of household education |  |  |  |  |  |
| No education | 46,7 | 36,3 | 49,3 | 59,4 | 49,5 |
| Primary | 29,2 | 44,3 | 32,5 | 31,9 | 32,9 |
| Secondary or higher | 24,1 | 19,4 | 18,2 | 8,6 | 17,6 |
| Household dietary diversity score (HDDS) |  |  |  |  |  |
| Low (0-4 food groups) | 67,5 | 66,9 | 76,6 | 85,5 | 74,2 |
| High (5-12 food groups) | 32,5 | 33,1 | 23,4 | 14,5 | 25,8 |

**Supplementary Table 2. Distribution of participants in the population and in the FGDs through arm of intervention and community type**

|  | **Study population** | | | **FGD** | | |
| --- | --- | --- | --- | --- | --- | --- |
|  | **SOC** | **SOC+NS** | **SOC+CT** | **SOC** | **SOC+NS** | **SOC+CT** |
| CSCD | 161 (35%) | 310 (64%) | 129 (27%) | 24 (38%) | 30 (49%) | 11 (18%) |
| FSCD | 106 (23%) | 11 (2%) | 131 (27%) | 20 (32%) | 5 (8%) | 26 (42%) |
| CSFD | 26 (6%) | 22 (5%) | 29 (6%) | 6 (10%) | 2 (3%) | 3 (5%) |
| FSFD | 170 (37%) | 138 (29%) | 190 (40%) | 13 (21%) | 24 (39%) | 22 (35%) |
| **Total** | 463 (100%) | 481 (100%) | 479 (100%) | 63 (100%) | 61 (100%) | 62 (100%) |

† CSCD, Close to services and distribution point; FGD: Focus Group Discussion; FSCD, Far from services but close to distribution point; CSFD, Close to services but far from distribution point; FSFD, Far from services and distribution point.

**Supplementary Table 3. Description of costs included in opportunity cost by intervention component.**

| **Type of cost** | **Costs included by intervention** | | |
| --- | --- | --- | --- |
|  | **Standard of care component** | **Nutrition supplementation component** | **Cash transfer component** |
| **Direct** | Transport costs to reach health post (kwanzas round trip)  Out of pocket costs | Transport costs to reach the distribution point (kwanzas round trip)  Out of pocket costs | Transport costs to reach the distribution point (kwanzas round trip)  Out of pocket costs |
| **Indirect** | Time to the health post (minutes on foot round trip) | Time to the distribution point (minutes on foot round trip) | Time to the distribution point (minutes on foot round trip) |
|  | Waiting time to receive medication (minutes) | Waiting time to receive the supplies (minutes) | Waiting time to receive the supplies (minutes) |
|  | Time spent in community sessions (minutes) |  |  |

† SOC, Standard of care; SOC+CT, Standard of care plus cash transfer; SOC+NS, Standard of care plus nutrition supplementation. SOC+NS intervention includes both the SOC component plus the Nutrition Supplementation component and and SOC+CT intervention includes the SOC component plus the Cash Transfer component.

**Supplementary Table 4. Median daily wages of community livelihoods.**

| **Livelihood** | **Median daily wage** | **Min** | **Max** |
| --- | --- | --- | --- |
| Agriculture | $1.90 | $0.96 | $7.02 |
| Casual selling of foods (bread, oil, tomatoes, onion, cookies) | $3.51 | $1.91 | $7.33 |
| Casual selling of traditional alcoholic drinks | $1.28 | $0.45 | $4.46 |
| Laundry | $1.91 | $0.29 | $6.38 |
| Selling coal | $0.99 | $0.38 | $1.59 |
| Domestic employee | $0.42 | $0.42 | $0.42 |
| **Total** | **$1.91** | **$0.29** | **$7.33** |

† All costs are in 2024 USD ($).

**Supplementary Table 5.** **Monthly opportunity cost in days of daily wage equivalent.**

|  | **SOC** | **SOC+NS** | **SOC+CT** |
| --- | --- | --- | --- |
|  | N days of work | N days of work | N days of work |
| **Total opportunity cost** | 0.88 | 1.47 | 1.66 |
| **Opportunity costs by community category** |  |  |  |
| Close to services and distribution point (CSCD) | 0.61 | 1.29 | 1.31 |
| Far from services but close to distribution point (FSCD) | 0.61 | 1.04 | 1.16 |
| Close to services but far from distribution point (CSFD) | 0.5 | 1.83 | 2.82 |
| Far from both services and distribution point (FSFD) | 1.37 | 1.83 | 2.06 |

† SOC, Standard of care; SOC+CT, Standard of care plus cash transfer; SOC+NS, Standard of care plus nutrition supplementation.
